# Acute Events in Primary Health Care Settings: An Analysis of Advanced Practice Competencies in Nursing Consultations in Brazil

**DOI:** 10.1002/nop2.70247

**Published:** 2025-07-21

**Authors:** Marília Orlandelli Carrer, Letícia Yamawaka de Almeida, Andrea Liliana Vesga‐Varela, Daiana Bonfim, Patrícia Aline de Almeida, Carla Pereira Barreto, Nayara Vilela de Farias Serranegra, Keila Gisele Lima Reis, Claudia Santos Martiniano, Manoel Vieira Miranda Neto

**Affiliations:** ^1^ Albert Einstein Israeli Faculty of Health Sciences São Paulo São Paulo Brazil; ^2^ Albert Einstein Israeli Hospital São Paulo São Paulo Brazil; ^3^ State University of Paraíba Campina Grande Paraíba Brazil; ^4^ School of Nursing, University of São Paulo São Paulo São Paulo Brazil

**Keywords:** advanced practice nursing, nursing process, primary health care, professional competence

## Abstract

**Aims:**

To describe nurse practices during acute event nursing consultations in Primary Health Care settings and analyse whether these professionals demonstrate the competencies proposed for Advanced Practice Nurses within a care management scope.

**Design:**

A multicenter, exploratory, cross‐sectional study employing both quantitative and qualitative analysis.

**Methods:**

Data were collected through direct non‐participatory observations (film footage of nursing consultations) and medical record analysis. The study included 28 nurses and 203 patients from 17 primary care units across four municipalities in Brazil. Descriptive statistical analysis was applied to quantitative data, while qualitative data underwent content analysis.

**Results:**

Challenges were observed in performing the nursing process and clinical communication during nursing consultations. Some of the proposed competencies for Advanced Practice Nurses were partially applied within a care management framework in Primary Health Care settings. The competencies related to the focus on care (61.11%), care provision (41.20%) and assessment and diagnosis (40.50%) were notable, with an overall average of 47.60% competency identification across all three dimensions. Although some general advanced practice nurse competencies were observed, their application was often limited or inconsistent.

**Conclusion:**

This study identified limitations in nursing consultations for acute events in Primary Health Care, particularly regarding the nursing process and the application of Advanced Practice Nurse competencies. Notable gaps were observed in areas such as cultural diversity and advanced assessment skills. Future efforts should focus on developing educational programmes, providing resources and creating clinical guidelines to enhance current nursing practices and facilitate the implementation of Advanced Practice Nursing in Brazil.

**Impact:**

The results provide empirical evidence to inform policy decisions, educational programmes, nursing practices and planning for advanced practice nursing implementation in Brazil. These findings are particularly relevant for healthcare educators, researchers, regulatory institutions and managers aiming to enhance the effectiveness of nursing consultations for acute events.

**Reporting Method:**

This paper adheres to the COREQ and STROBE checklist.

**Patient or Public Contribution:**

No patient or public contributions.

## 1 Introduction

Demographic and epidemiological population profile changes worldwide require the reorganisation of current health systems, with a strategic emphasis on strengthening Primary Health Care (PHC) and expanding the competencies of PHC professionals (Oliveira et al. 2018; Cassiani and Silva 2019; PAHO 2018), aiming at improving health access and universal health coverage (Cassiani and Silva 2019). In this context, the implementation of Advanced Practice Nursing has emerged as an innovative solution, particularly within PHC settings, being recognised and encouraged by the Pan American Health Organization (PAHO/OMS) as a strategy to expand nursing roles and responsibilities (PAHO 2018), ultimately enhancing patient access and satisfaction within health services across Latin America and the Caribbean (Cassiani et al. 2018). This is especially relevant given that nursing represents the largest workforce category in the healthcare sector. However, Advanced Practice Nurse (APN) remains an unrecognised professional role in most Latin American countries, including those with significant advancements in nursing, such as Brazil.

An APN integrates research, education, clinical practice and management skills to autonomously conduct health assessments, make diagnoses, prescribe treatments and manage both acute and chronic conditions. They also implement care plans, facilitate referrals, serve as key contacts for health service users and develop additional competencies related to leadership, collaboration and academic expertise in evidence‐based practice. These competencies are recognised as essential for providing person‐centred care, conducting effective health assessments and working collaboratively with patients, families and multidisciplinary teams (Søndergaard et al. 2024).

According to the International Council of Nurses (ICN), an APN is defined as:

[…] nurses who have acquired a specialized knowledge base, complex decision‐making skills and clinical competencies for expanded practices, whose characteristics are shaped by the context and/or country in which they are licensed to practice. A master's degree is recommended for entry level (ICN 2020).

Although Brazil lacks formal training and regulations for APN to date, discussions regarding the implementation of Advanced Practice Nursing have progressed, driven by institutions such as the Federal Nursing Council (*Conselho Federal de Enfermagem*, COFEN). This council has not only promoted research on the subject to support the development of an APN competency model in the country, but also recently published a technical note 001/2023, entitled ‘Advanced Practice Nursing in Brazil: context, concepts, actions taken, implementation and regulations’ (COFEN 2023).

The topic has garnered considerable attention as a potential solution for reducing disparities in access to quality healthcare and addressing existing deficiencies, as evidence from countries where the APN role has been implemented shows not only increased access to services and the resolution of health issues, but also improvements in user satisfaction, cost reductions for governments and greater autonomy for nurses in their clinical practice (Spinoza and Toso 2021).

However, while discussions are advancing, significant challenges persist in understanding the practicalities of integrating APNs into Brazil's PHC system. Despite well‐defined international guidelines, there is a lack of empirical evidence exploring the specific obstacles and learning opportunities for nurses, as well as clarity on the core competencies required for APNs considering the necessary adaptations for effectively implementing these roles in Brazil's diverse healthcare settings.

## 2 Background

Access to healthcare in Brazil primarily occurs through the Unified Health System (SUS), which is offered as a universal service, organised in a polyarchic network across different levels of care: PHC, secondary care (specialty outpatient clinics and medium‐sized hospitals) and tertiary care (high‐complexity hospitals and reference centers).

Brazilian PHC is considered promising for the implementation of advanced nursing roles, as Registered Nurses (RN) are seen as key figures in the country and already demonstrate some of the proposed APN skills (Gomes et al. 2024; Almeida et al. 2021), with Nursing Consultations (NC) as the primary tool for care. NC are regulated by COFEN resolution 736/2024, which provides for nursing processes carried out in five interrelated, interdependent and recurring stages, namely assessment, diagnosis, planning, implementation and evolution (COFEN 2024).

To effectively perform NC, RN must integrate skills grounded in scientific knowledge, nursing theories and ethical and human aspects associated with communication techniques, enabling them to provide comprehensive care beyond a problem‐oriented approach when developing clinical reasoning and diagnostic judgements (COFEN 2024).

Given this complexity, the need for a clear competency model in PHC becomes evident. A competency model is understood as attitudes developed through previously acquired knowledge, coupled with the development of skills and concern for others employing awareness and initiatives according to the professionals' responsibility field (Zarifian 2003).

In the Brazilian PHC context, NC addresses both acute and chronic healthcare demands. However, despite advancements in comprehensive primary care that ensure first‐contact access for users, managing these demands—both acute and chronic—remains a significant challenge for the healthcare system. Thus, the search for effective strategies to organise the provision of care for the increasing demand for acute events (AE) in PHC—while ensuring access, maintaining system efficiency and preventing the overburdening of hospitals and emergency departments with cases that could be managed within primary care—is an ongoing concern for healthcare managers (Mendes 2019).

Acute demands typically last up to 3 months, are self‐limiting, have a sudden onset and respond well to specific treatments (Mendes 2019; Ministry of Health, Brazil 2013). Clinical nursing practices in this setting are generally supported by protocols and clinical guidelines, which enhance their effectiveness (Ministry of Health, Brazil 2013). Thus, considering the discussions and scientific evidence on the potential of the APN in the healthcare system, the implementation and development of this role could strengthen the system's ability to provide effective and timely care in PHC. Therefore, this study aims to address the following research question: What are the competencies already developed by RNs in PHC services, and how do they compare to the expectations for Advanced Practice Nursing, based on a competency model proposed for Latin American and Caribbean countries? (Cassiani et al. 2018).

## 3 The Study

### 3.1 Aims

Describe RN practices during AE nursing consultations in PHC settings and analyse whether these professionals present APN‐proposed competencies within a care management scope.

## 4 Methods

### 4.1 Design

This study is a multicenter, exploratory, cross‐sectional assessment employing both quantitative and qualitative analyses. It is part of the matrix project entitled ‘Advanced practice competencies in care management in primary care nursing consultations: situational diagnosis and training proposal’, which aims to evaluate NC in different PHC lines of care. This article will present data concerning the investigation of NC attention to AE.

It is noteworthy that the reporting of this study was guided by Consolidated Criteria for Reporting Qualitative Research (COREQ) and Strengthening the Reporting of Observational Studies in Epidemiology (STROBE) guidelines (Souza et al. 2021; STROBE 2023).

**FIGURE 1:**
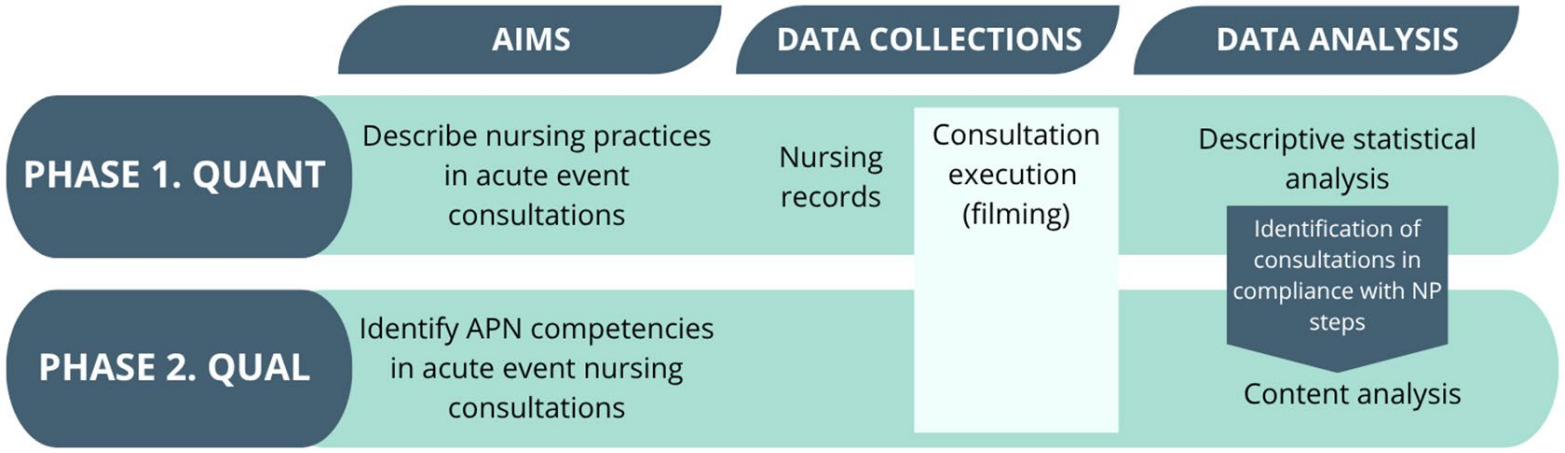
Description of the methodological stages of the study. Brazil, 2022.

The study stages are outlined in Figure 1, which presents the objectives, data collection methodology and data analysis for both phases of the study: quantitative and qualitative.

### 4.2 Study Setting and Sampling

This study was conducted in 17 Primary Care Units (PCU) located across four Brazilian municipalities: four in municipality A (state of São Paulo), six in municipality B (state of Amazonas), two in municipality C (state of Alagoas) and five in municipality D (state of Rio Grande do Norte), representing a total of 54 Family Health Strategy teams.

The sample size and design were determined using convenience sampling, a non‐probability sampling method in which participants are selected based on their availability and accessibility (Shorten and Moorley 2014). This approach was used to encompass diverse geographic and demographic realities across Brazil. Three of the country's five regions were covered, including two large municipalities (A and B) and two smaller ones (C and D), ensuring a broad range of contexts related to demographic, morbidity, mortality and service–offering profiles.

The choice of convenience sampling was driven by practical limitations such as time, resources and access to the health units included in the study. Data collection was pre‐arranged with the management and nursing teams, and efforts were made to minimise any interference with the daily care routine, as the RNs were performing their professional duties. Moreover, it was not possible to predict which health demands would be addressed on any given day, nor whether patients would consent to participate in the study. Therefore, despite potential selection biases, convenience sampling was deemed the most suitable approach for this research.

### 4.3 Inclusion and/or Exclusion Criteria

The inclusion criteria for RNs were employment in a participating PCU and active involvement in direct patient care for at least 1 year. No additional qualifications beyond a nursing degree were required, as the study aimed to explore the dynamics of NC for AE across various PHC settings, without restricting participation to those with specialised training. Exclusion criteria included professionals on leave or vacation and nurses not directly involved in patient care, such as those in management roles.

The inclusion criteria for the NC required attendance due to AE in PHC settings, and the video footage of the NC had to be complete for data analysis. In other words, the recording needed to capture the entire consultation, from start to finish, with clear image and sound quality.

### 4.4 Data Collection

Data collection took place between May and July 2022. Following an invitation and agreement with the PCU management and participating RN, an Informed Consent Form (ICF) was obtained from those who agreed to participate. Additional instruments were also used, including a form that authorised the use of RNs' images and voices, as well as instruments for nursing characterisation. These instruments collected sociodemographic data, professional training background, field characteristics and professional motivations.

The data collection was carried out by two researchers. The first researcher approached patients in the waiting room, providing an explanation of all relevant terms, including the project proposal and the data collection method involving videotaping. If patients consented to participate, they were given the necessary forms, including the ICF, an authorization form for the use of images and voice, and a patient characterisation instrument. This instrument collected sociodemographic data, personal history, reasons for seeking nursing care, the preferred healthcare professional for PHC care, and satisfaction with the nurse's care. Upon accepting and signing the terms, the first researcher identified participants using coloured bracelets (blue and orange) to allow the second researcher to recognise that the patient had agreed to participate.

The second researcher managed the recording process using two GoPro Hero9 cameras. One camera was placed in the office to capture both sound and images of all interactions between RNs and patients during the consultation. The other camera, fixed to the nurse's body with an elastic chest strap, focused on physical examinations and situations in which the nurse was absent from the office, such as when discussing cases with other health professionals. The researcher activated the cameras at the beginning of the consultation, presented the user's identification code (consisting of the patient's initials, followed by the numerical order of the consultation and the initials of the municipality), and positioned the lapel mic to capture sound. After ensuring that the cameras were functioning properly, the researcher left the room and returned only after the consultation to turn off the equipment. To ensure secure data storage, institutional cloud software with a robust protection system was used, with exclusive access for project researchers.

Consultation archives were obtained from physical or electronic medical records after the end of the NC using a researcher checklist. Instrument development and data management were carried out in electronic format using the RedCap platform (Research Electronic Data Capture) (Harris et al. 2019).

### 4.5 Data Analysis

#### 4.5.1 Quantitative Analysis

At this stage, nursing practices concerning NC and AE were described by the film footage and medical records based on the researcher‐prepared instruments.

All videos were analysed using a semi‐structured script, in which the presence or absence of nursing process steps was highlighted. COFEN resolution no. 358/09 (COFEN 2024), in force during the data preparation, collection and analysis period of this study, as well as the Brazilian Ministry of Health Basic Care Booklet number 28 volumes I and II (Ministry of Health, Brazil 2013), person‐centered medicine concepts (Stewart et al. 2017); and the communication skills guide ‘Calgary‐Cambridge Guide to the Medical Interview—Communication Process’ were employed (Kurtz et al. 2003). A checklist was also prepared and used to analyse nursing records, identifying the presence or absence of nursing process steps, which were systematically organised according to the SOAP model elements: Subjective, Objective, Assessment and Plan (Queiroz 2009).

A descriptive analysis employing central tendency, dispersion and frequency measures was carried out for all variables. Variable distributions were assessed using the Kolmogorov–Smirnov test, and differences between groups were evaluated by the chi‐squared or Fisher test, when necessary, and a *p*‐value < 0.05 was adopted to identify statistically significant associations. A bivariate analysis was graphically carried out to generate possible hypotheses regarding nursing process compliance.

Adherence to the nursing process was calculated by adjusting the number of items evaluated in each step, ensuring equal weight for each item in the overall compliance percentage. The calculated adherence percentages were then presented graphically, according to patient, nurse and consultation characteristics. NC with adherence to the nursing process of ≥ 50% were chosen for an in‐depth analysis of the competencies proposed for APN in PHC settings. All statistical analyses were conducted with the assistance of statistical support using R statistical software (version 4.1.3, R Project for Statistical Computing) and RStudio (version 1.4.1717).

#### 4.5.2 Qualitative Analyses

This stage aimed to investigate whether RN demonstrated the care management competencies proposed for APN during NCs related to AE. A content analysis (Bardin 1977) was performed to explore this, with data interpretation guided by categories derived from the care management competency model for Latin American and Caribbean APNs in PHC settings, as outlined by Cassiani et al. (2018). This model is based on a three‐dimensional framework: care focus, assessment and diagnosis, and care provision, incorporating 20 competencies (Cassiani et al. 2018).

The content of the footage was systematically examined, and excerpts reflecting the presence or absence of the competencies were extracted, including environmental, dialogical and attitudinal elements that enriched the qualitative analysis. The lead author transcribed all relevant material, ensuring accuracy and preserving the integrity of the data.

An inductive content coding process followed, with the identification of excerpts corresponding to aspects of the three‐dimensional framework. This phase involved two trained analysts working collaboratively, followed by meetings to reach consensus on the identified codes. The data were then grouped into categories (20 competencies) based on similarity and meaning, guided by the adopted theoretical framework and relevant literature. The categorization process involved multiple rounds of review and discussion to ensure that contextual and cultural nuances were captured.

Finally, analytical generalisation was employed to identify patterns and insights that could contribute to a broader understanding of the phenomenon. The frequency of occurrences for each category was also reported. To ensure confidentiality and adhere to ethical standards, participants' identities were anonymized using alphanumeric codes in the transcriptions. These procedures ensured the validity and reliability of the study, accurately reflecting the interactions observed during the NC.

The report of the qualitative approach of this study was conducted in accordance with the COREQ guidelines (Souza et al. 2021), which helped structure and enhance its rigor.

### 4.6 Ethical Considerations

This study was approved by the REDACTED Ethics Committee (CAAE: 56255622.2.0000.0071), with the consent of all co‐participating centres. Participation was conditional on Informed Consent Form completion and signing.

## 5 Findings

The findings encompass the observation of 58 NC at AE carried out by 24 RNs, distributed throughout municipalities A (63.2%), B (26.3%) and D (10.5%). It is important to note that no NC with AE reasons was identified for municipality C. The results were organised detailing patient descriptions and identifying health demands, RNs profiles, nursing consultation overviews based on the film footage, nursing record details extracted from medical records and, finally, descriptions of APN‐proposed care management competencies.

The patients attending NC had a mean age of 37 years (SD ± 18.5), with a majority being female (65.2%) and self‐identifying as mixed racial heritage (pardo) (61.4%). Additionally, 26.32% self‐identified as caucasian, and 10.53% as black. More than half had completed high school (53.8%), while a significant portion were unemployed (42.3%) and did not have partners (50%). Additionally, most had children (78.9%) and a family income of up to two minimum wages (74.1%) per month, with the Brazilian minimum wage in 2022 set at approximately US$ 252.00 (R$ 1212.00).

The International Classification of Primary Care system—2nd Edition (CIAP‐2) was used concerning the patients' health demands (WHO 2009). The most frequent demands concerned the female genital system (20%), followed by the musculoskeletal system (19%), digestive system (12%) and skin (11%).

Among the RNs who performed NC for AE, 11 (45.8%) were from southeastern Brazil, while 13 (54.2%) were from the northern and northeastern regions. Their mean age was 39.86 years (SD ± 7.35). The majority were female (78.3%) and worked in mixed or exclusively Family Health Strategy (FHS) units. Similarly to the patient group, most RNs (52.2%) self‐identified as having mixed racial heritage (Pardo), and although their salaries varied, the majority earned more than seven minimum wages per month. Moreover, 56.5% did not have a second job.

When it comes to professional experience, 36.4% of the RNs had 1 to 5 years of experience as family nurses, another 36.4% had 6 to 10 years in this role, and 52.2% had more than 10 years of experience. Most of them worked in PCUs with multidisciplinary teams (65.2%), and the use of electronic medical records was widespread (91.3%). Furthermore, 69.6% had their own consulting rooms, which eliminated the need to share space with other professionals.

Despite feeling satisfied with their work (52.2%), the majority of RNs reported challenges in conducting their consultations (65.2%). They did not use standardised tools for the nursing process (60.9%) and employed a variety of protocols in their consultations. In terms of education, most RNs obtained their bachelor's degree from public universities (52.2%), 87% studied the nursing process, and 95.7% had postgraduate qualifications.

More detailed characteristics of the participating RNs are presented in Table 1, classified into two groups: compliance ≥ 50% with nursing process steps (including those who completed more than 50% of the nursing process steps during the analysis of their NC) and compliance < 50% with nursing process steps (including those who did not achieve at least 50%).

**TABLE 1:** Registered nurses sociodemographic and work characteristics according to the nursing process steps completion score (*n* = 24). Brazil, 2022.

Variables	Compliance with the nursing process steps		
	< 50%, *n* (%)	≥ 50%, *n* (%)	Total, *n* (%)
Race			
Caucasian	3 (30)	6 (46.2)	9 (39.1)
Mixed Racial Heritage (Pardo)	7 (70)	5 (38.5)	12 (52.2)
Asian	0 (0.0)	2 (15.4)	2 (8.7)
Individual income			
Up to 2 MW^a^	0 (0.0)	0 (0%)	0 (0.0)
3 to 4 MW^a^	2 (20)	2 (15.4)	4 (17.4)
5 to 6 MW^a^	3 (30)	0 (0.0)	3 (13.0)
Over 7 MW^a^	5 (50)	11 (84.6)	16 (69.6)
Other employment relationships			
Yes	5 (50.0)	5 (38.5)	10 (43.5)
No	5 (50.0)	8 (61.5)	13 (56.5)
Experience as a nurse			
Between 1 and 5 years	1 (10.0)	1 (7.7)	2 (8.7)
Between 6 and 10 years	3 (30.0)	6 (46.2)	9 (39.1)
Over 10 years	6 (60.0)	6 (46.2)	12 (52.2)
Is there a multidisciplinary team at the health unit?			
Yes	4 (40.0)	11 (84.6)	15 (65.2)
No	6 (60.0)	2 (15.4)	8 (34.8)
Experience as an FHS nurse			
Less than 1 year	1 (10.0)	0 (0.0)	1 (4.5)
Between 1 and 5 years	4 (40.0)	4 (33.3)	8 (36.4)
Between 6 and 10 years	4 (40.0)	4 (33.3)	8 (36.4)
Over 10 years	1 (10)	4 (33.3)	5 (22.7)
Is there an electronic medical record available in the unit?			
Yes	9 (90.0)	12 (92.3)	21 (91.3)
No	1 (10.0)	1 (7.7)	2 (8.7)
Is there an exclusive room available for nursing consultations?			
Yes	8 (80.0)	8 (61.5)	16 (69.6)
No	2 (20.0)	5 (38.5)	7 (30.4)
Are difficulties carrying out a consultation noted?			
Yes	6 (60.0)	9 (69.2)	15 (65.2)
No	4 (40.0)	4 (30.8)	8 (34.8)
Are standardised nursing process instruments available?			
Yes	3 (30.0)	6 (46.2)	9 (39.1)
No	7 (70.0)	7 (53.8)	14 (60.9)
Nurse motivation scale			
Dissatisfied	0 (0.0)	1 (7.7)	1 (4.3)
Neither satisfied nor dissatisfied	1 (10.0)	0 (0.0)	1 (4.3)
A little satisfied	0 (0.0)	3 (23.1)	3 (13.0)
Satisfied	6 (60.0)	6 (46.2)	12 (52.2)
Very satisfied	3 (30.0)	3 (23.1)	6 (26.1)
Type of university (bachelor's degree)			
Public	6 (60.0)	6 (46.2)	12 (52.2)
Private	4 (40.0)	7 (53.8)	11 (47.8)
Has finished a postgraduate course			
Yes	9 (90.0)	13 (100)	22 (95.7)
No	1 (10.0)	0 (0.0)	1 (4.3)
Type of postgraduate course			
Primary Health Care Specialisation	0 (0.0)	6 (46.2)	6 (26.1)
Family and Community Health, Collective Health or Public Health Specialisation	4 (40.0)	7 (53.8)	11 (47.8)
Professional master's degree	0 (0.0)	2 (15.4)	2 (8.7)
Academic master's degree	1 (10.0)	1 (7.7)	2 (8.7)
Other	7 (70.0)	6 (46.2)	13 (56.5)
Took classes on the nursing process			
Yes	10 (100.0)	10 (76.9)	20 (87)
No	0 (0.0)	1 (7.7)	1 (4.3)
Does not know	0 (0.0)	2 (15.4)	2 (8.7)
Are nursing protocols used in the operating unit?			
Yes	5 (50.0)	11 (84.6)	16 (69.6)
No	5 (50.0)	2 (15.4)	7 (30.4)
Protocols used for acute events			
Ministry of Health Basic Care Booklet	6 (60.0)	9 (69.2)	15 (65.2)
Ministry of Health Protocol	5 (50.0)	7 (53.8)	12 (52.2)
State Protocol	0 (0.0)	1 (7.7)	1 (4.3)
Protocol for other municipalities	2 (20.0)	1 (7.7)	3 (13.0)
Regional Nursing Council Protocol	0 (0.0)	0 (0.0)	0 (0.0)
Protocol from the municipal health department	2 (20.0)	7 (53.8)	9 (39.1)
Others	0 (0.0)	1 (7.7)	1 (4.3)

Abbreviation: FHS, Family Health Strategy.

^a^ MW = Current minimum wage per month, Brazil, 2022 (US$ 252.00 or R$ 1212.00).

Regarding NC, approximately 80% (46) of the demands associated with AE comprised the main consultation reason, being the secondary reason in the rest of the cases, and were expressed after RNs questioning or when identified in physical examinations. The average NC time was 20.22 min (SD ± 15.47), with a high percentage of consultation interruptions (SD **≅** 52.63%) due to requests for RNs or other reasons.

During the nursing assessment phase, most RNs conducted data collection based on the patient's acute needs, appropriately prioritising them. However, not all of them explored the underlying causes or integrated the patient's medical history into their clinical reasoning. Additionally, there was limited attention to the patient's emotions, fears, thoughts and expectations during this stage. It is also important to note that all the cases analysed required a physical examination, which was performed by 76.19% of the RNs.

In the diagnosis phase, only 31.03% of the RNs formulated a nursing diagnosis, and just 48.28% developed a care plan. Furthermore, 31.03% issued a nursing prescription. It is noteworthy that, in the care implementation stage, almost all RNs applied interventions directly related to the patient's acute complaint.

In addition, according to the expert's opinion, the involvement of additional healthcare professionals was necessary to address the patient's complaint in 74.14% of cases. However, only 43.10% of patients were referred to a physician, and an insignificant percentage were referred to the interprofessional team or to other services within the Urgency and Emergency Care Network.

Table 2 presents a detailed nursing process steps compliance description of the 58 NCs conducted in PHC settings.

**TABLE 2:** Description of nursing process compliance steps in nursing consultations in Primary Health Care settings. Brazil, 2022.

Nursing process steps	Absent	Present
	*n* (%)	*n* (%)
Nursing assessment		
Did the RN conduct the data collection based on the patient's acute demand?	18 (31.03)	40 (68.97)
Did the RN investigate the causes related to the acute demand?	30 (51.72)	28 (48.28)
Did the RN consider the individual's clinical history in their clinical reasoning to meet the acute demand?	26 (44.83)	32 (55.17)
Did the RN assess whether the acute event is related to a chronic or recurrent condition?	20 (34.48)	38 (65.52)
If yes to the previous question, is it an exacerbated chronic condition?	29 (76.32)	09 (23.68)
Did the RN listen to/value the patient's demand in relation to the acute event?	24 (41.38)	34 (58.62)
Did the RN dedicate him/herself to understanding the patient's feelings and fears about his/her demands?	52 (89.66)	06 (10.34)
Did the RN dedicate him/herself to understanding the patient's ideas about what is wrong?	44 (75.86)	14 (24.14)
Was the RN dedicated to understanding the effects of the disease on the patient's functions?	49 (84.48)	09 (15.52)
Did the RN dedicate him/herself to understanding the patient's expectations regarding their care?	46 (79.31)	12 (20.69)
Did the RN perform a physical examination during the consultation? (may be partial)	16 (27.59)	42 (72.41)
Does the reported or identified acute event require a specific physical examination?	00 (0.00)	42 (100.0)
Did the RN perform a physical examination related to the acute event?	10 (23.81)	32 (76.19)
Nursing diagnosis		
Did the RN reach a nursing diagnosis?	40 (68.97)	18 (31.03)
Planning		
Did the RN agree on the care plan?	30 (51.72)	28 (48.28)
Did the RN hand out a nursing prescription?	40 (68.97)	18 (31.03)
Implementation		
Did the RN apply intervention/care elements associated with the acute event complaint?	05 (8.62)	53 (91.38)
In the expert's opinion, was it necessary to include the attention of other health professionals to address the patient's complaint?	15 (25.86)	43 (74.14)
Did the RN refer the patient to the doctor?	33 (56.9)	25 (43.10)
Did the RN forward the patient to the multidisciplinary team?	54 (93.1)	04 (6.90)
Did the RN carry out an interconsultation?	51 (87.93)	07 (12.07)
Did the RN carry out a shared consultation?	54 (93.10)	04 (6.90)
Did the RN encourage a search for longitudinal care?	29 (50.0)	29 (50.0)
Did the RN carry out referrals to other urgency and emergency care network points?	57 (98.28)	01 (1.72)
Did the RN provide guidance on alarm signs or when to seek the service again?	45 (77.59)	13 (22.41)
Did the RN provide guidance on the pathophysiology and evolution of the health problem?	45 (77.59)	13 (22.41)
Nursing evolution		
Did the RN mention the need for a new meeting?	23 (39.66)	35 (60.34)
Did the RN correctly apply the risk concept associated with the acute event?	12 (20.69)	46 (79.31)
Considering the risk concept, was the intervention appropriate?	13 (22.41)	45 (77.59)
Is this a return appointment or longitudinal follow‐up appointment?	42 (72.41)	16 (27.59)

Abbreviation: RN, registered nurses.

**FIGURE 2:**
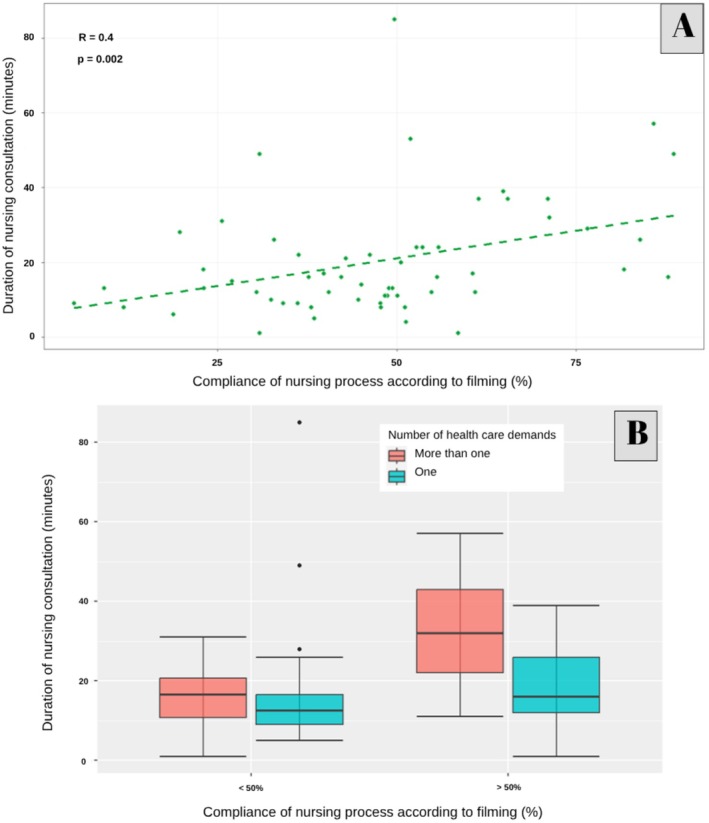
Assessment of consultation times and nursing process step compliance. Brazil, 2022. (A) Correlation between nursing consultation times and nursing process step compliance percentages; (B) Description of median consultation times according to nursing process step compliance percentages and number of demands.

A positive and weak correlation was identified between NC times and compliance with nursing process steps (*R* = 0.40, *p* = 0.002) (Figure 2A). The duration of AE consultations was longer among RNs who completed over 50% of the nursing process steps, and even longer in consultations in which the user presented more than one demand (Figure 2B).

Besides analysing nursing process steps, aspects concerning clinical communication (Kurtz et al. 2003) were also evaluated, focusing on how RNs interact with patients both verbally and non‐verbally. The following observations were made during the NC: whether the RN paid attention to the patient's comfort/privacy (91%), adapted their language to avoid scientific jargon (95%), refrained from criticism or value judgements about the patient (93%), and avoided non‐verbal communication that denoted disinterest or disapproval (91%).

However, other aspects were not frequently observed, such as RN introduction (22%), repeating the patient's message and validating their understanding and information (25%), verifying the patient's understanding and encouraging doubt clarification (36%), requesting feedback on the patient's understanding of the covered content (9%), encouraging the verbalisation of feelings, expectations and/or concerns (22%) and summarising information and prioritising demands (3%).

Regarding nursing records, 53 consultations (91.37%) were documented, 50 of which were available as electronic records. All records were dated, 92.45% reported the consultation time, and the SOAP method (Queiroz 2009) was used as a record standardisation instrument in most cases (92.45%).

Concerning the nursing process steps analysed in the records, at the assessment stage, subjective data were fully recorded in 37.74% (20) of the records and partially recorded in 54.72% (29). Objective data were fully recorded in only 5.66% of the records and partially recorded in 84.91%, while physical examinations were absent in 24.53% of the records.

Nursing diagnoses were present in 58.49% of the analysed records. Of these, only 30.19% completely represented the findings obtained during the assessment stage. At least one CIAP‐2 was present in all electronic medical records (94.34%) and the CIAP‐2 (WHO 2009) was associated with the assessment stage in 81.13% of the cases.

Concerning the planning stage, goals and expected outcomes were absent in 86.79% of all cases. Only 11.32% of the records fully documented the nursing prescription, and just 22.64% of the prescriptions were either fully or partially aligned with the nursing diagnosis. The implementation stage was absent in 96.23% of the records, while the evolution stage was not documented in 81.13% of cases. However, it is important to note that the evolution did not apply to 71.70% of the cases, as they were first‐contact consultations for the addressed health conditions.

When analysing the description of APN‐proposed care management competencies among the 58 NC included in the sample, 24 that complied with 50% or more of the nursing process steps were selected for a qualitative analysis to assess the presence of these competencies in PHC settings (Cassiani et al. 2018).

The 24 NC were carried out by 13 RNs, eight from municipality A, four from municipality B and one from municipality D. The most frequently addressed health demands according to the CIAP‐2 classification were as follows: X14 Vaginal secretion (7), D06 Other localised abdominal pain (3), N01 Headache (3) and R05 Cough (3). It is important to note that two or more patient demands were addressed in eight of the consultations.

The RNs who carried out NC for AE partially applied some APN‐proposed skills in PHC settings within a care management scope. Focus on care dimension (61.11%), care provision (41.20%) and assessment and diagnosis (40.50%) were all noteworthy, with an overall average of 47.60% competency identification considering the same weight for all three dimensions.

The evidence extracted from the film footage (scenes and speech) that best represents the expected APN care management competencies and their frequencies is summarised in Table 3.

**TABLE 3:** Summary of the proposed Advanced Practice Nurse competencies in care management in Primary Health Care settings present in acute event consultations. Brazil, 2022.

Theme	Description of the scene and speech taken from nursing consultations	Competencies proposed for EPA in PHC (4)	Freq
Focus on care	Consultation: 1807‐02 Scene: The nurse investigated the patient's history, interspersing looking at the computer and at the user. In a moment, the nurse gets up, arranges the stretcher and invites the patient to lie down. She continues to ask questions while performing the physical exam Speech: *On Thursday, a discharge began coming out of my belly button, out of nowhere. It had already happened once and then it stopped. It came out on Thursday and Friday and on Saturday it didn't go away, there was just that unpleasant smell* (P23); *Did you have a fever?* (RN21); *Nothing* (P23); *Have you had any belly surgery?* (RN21); *I had a hidrolipo, but it's already been a year* (P23); *And was it through the belly button?* (RN21); *No, the cutout was here on the side. What I felt on Wednesday night was a cramping pain, very strong, so I put on that hot pack* (P23); *In addition to this surgery, have you ever had any other abdominal surgery?* (RN21); *Only a c‐section, the last one is 7 years old* (P23); *Do you have any health problems or allergies to any medication? Did you have any other symptoms?* (RN21); *No. Another issue that bothers me a lot is haemorrhoids. A while ago I came here, the doctor gave me some ointment, but sometimes it comes out, I wanted to see if it was possible to perform microsurgery to remove it* (P23); *Yes, you need to examine it to see how it is, to be forwarded. And what is your diet like?* (RN21); *I eat everything, except pepper, which I know makes it worse* (P23); *Do you eat vegetables every day?* (RN21); *No, only from time to time, I'm not a big fan* (P23); *How does your intestine work?* (RN21); *Very well, every day, 3 to 4 times a day* (P23); *And is this your normal? Does it come out pasty?* (RN21); *Yes. Normal* (P23); *Today what bothers you most is the belly button issue, right?* (RN21); *Yes* (P23); *And from that part of the hemorrhoid, isn't it protruding or bleeding?* (RN21); *No* (P23); *Does the secretion come out when you are sitting or standing?* (RN21); *No, I went to work and when I came back at night, I was about to take a shower I saw that it was wet* (P23); *Did your belly feel hard, swollen?* (RN21); *Nothing* (P23); *I'm going to talk to the doctor, and I'll be right back* (RN21) (Leaves the room to carry out a consultation)	Incorporates cultural diversity and health determinant knowledge into the patient's assessment, diagnosis and therapeutic management, as well as in the result evaluation	37.5% (9)
		Incorporates knowledge concerning development and life stages, pathophysiology, psychopathology, epidemiology, environmental exposure, infectious diseases, behavioural science and demographics and family processes when performing assessments, making diagnoses and providing therapeutic management	54.17% (13)
		Incorporates knowledge on the clinical manifestations of normal health events, acute illnesses/injuries, chronic illnesses, comorbidities and health emergencies, including the effects of multiple etiologies, in the assessment, diagnosis, therapeutic patient management and outcome evaluation	91.7% (22)
Assessment and Diagnosis	Consultation: 2607‐07 Scene: The patient is lying on the stretcher for a physical examination. The nurse receives a doctor and a medical student in the room to carry out a consultation and gives the doctor a summary of the condition and what she thought after her assessment Speech: *Doctor, this is (name), a construction worker, he has had pain for 7 days in the inguinal region, he went to AMA (Outpatient medical care) on Saturday, and was treated with some injections, which he can't name. I examined him, and after the manoeuvre that I asked him to do, I palpated bilaterally, and I found that the area is very enlarged and painful. I will ask for some quick tests too, there is nothing in the urethra, there is no secretion, the penis is undamaged, but I will test that too. We need to reach a differential diagnosis, whether it is a hernia, or an infection. I'll call the girls in a little while so we can do quick tests* (nursing techniques) (RN31)	Adapts interventions to respond to the needs of people and families in aging, in life transitions, in comorbidity situations, and considering psychosocial and financial situations	8.3% (2)
		Uses technological systems to collect data on variables concerning patient evaluations	9.1% (2)
		Accurately collects and documents relevant patient history at each life stage and family life cycle, using other collateral information if necessary	33.3% (8)
		Accurately performs and documents appropriate or symptom‐focused physical examinations in patients of all ages (including developmental and behavioural screenings, physical exams and mental health assessments)	58.33% (14)
		Identifies health and psychosocial risk factors for patients of all ages and families at all family life cycle stages	37.50% (9)
		Performs differential diagnoses between acute, chronic and life‐threatening conditions	87.50% (21)
		Plans screening and diagnostic strategies making appropriate use of technology as a tool, considering client costs, risks and benefits	45.83% (11)
Provision of Care	Consultation: 2107–05 Scene: A nurse receives a patient in the office to collect a pap smear and identifies the presence of a vaginal discharge during the procedure, after which she seats the patient again in a chair next to her, and provides guidance regarding treatment and follow‐up care, making eye contact and gesturing Speech: *I will give you miconazole, which is a vaginal cream, which you will use for 10 nights. During these 10 nights, do not have sex, take a shower and apply the ointment* (E24); *But I think my period is about to start* (P32); *So you can wait, when it's done finish you start, so we don't interrupt it in the middle, don't stop. But then use the ointment. You can get the pap's result from me after 30 days. Blood tests, you haven't had those in a while, right?* (RN24); *Right* (P32); *I'll ask for those too. Are you 18?* (RN24); *No. But I had gestational diabetes in my last pregnancy two years ago* (P32) (…) *Panties, preferably not washed in the bathroom and not left in the bathroom. OK? Dry them in the sun, outside, if you can't dry them in the sun, at least iron the bottom of your panties, to avoid bacteria. Wash them with soap or coconut soap and preferably wash them separately from other clothes* (RN24); *Yes, we wash everything together, with fabric softener* (P32); *If that doesn't solve this, then we'll look at the results and reevaluate, and treat it again, and see if there are other pap findings* (RN24)	Provides consistent care in accordance with what is established in clinical guidelines and protocols	54.55% (12)
		Provides care in a manner that respects and promotes cultural diversity	0.0%
		Communicates effectively, addressing clinical findings, diagnosis and therapeutic interventions	50% (12)
		Determines care options and formulates a therapeutic plan in collaboration with patients, considering their expectations and beliefs, available evidence and the cost–benefit ratio of interventions	45.83% (11)
		Incorporates the principles of quality and patient safety into clinical practice	41.67% (11)
		Begins a therapeutic plan, carrying out pharmacological and non‐pharmacological interventions, treatments or therapies	95.83% (23)
		Prescribes medications within its scope of action (national regulations and protocols/programs)	69.23% (9)
		Monitors the patient's progress, evaluating and adjusting the therapeutic plan according to their responses	62.5% (5)
		Adapts interventions to respond to the needs of people and families in aging, in life transitions, in situations of comorbidity, and considering psychosocial and financial situations	25% (6)
		Develops an appropriate palliative and end‐of‐life care plan	Does not apply

In the first dimension, focus on care, a notable positive finding is the competency related to the knowledge of clinical manifestations of normal health events, acute illnesses/injuries, chronic conditions, comorbidities and health emergencies, which was present in 91.7% (22) of the consultations analysed, though in a partial manner.

As seen in the scene and speech excerpt presented for this dimension, the RN leads the consultation by asking questions that investigate the origin of the health issue presented by the patient, correlating it with their health history, habits and ruling out alarm signs, demonstrating knowledge of clinical manifestations.

In the second dimension, assessment and diagnosis, the competency that stands out most positively is the ability to perform differential diagnoses between acute, chronic and life‐threatening conditions, which was observed either fully or partially in 87.5% (21) of the consultations. In contrast, competencies related to adapting interventions based on the unique needs of individuals and families, as well as the use of technologies in care, were scarcely represented, appearing in only 8.3% (2) and 9.1% (2) of the consultations, respectively.

In the excerpt that is illustrated for this dimension, we observe a RN discussing a case with a physician. In her speech, she presents key elements of her assessment stage, explaining her clinical reasoning, outlining diagnostic hypotheses and recommending a complementary exam to be conducted at that moment to complement the findings. This demonstrates competencies such as performing appropriate or symptom‐focused physical examinations; planning screening and diagnostic strategies while making appropriate use of technology as a tool; and performing differential diagnoses between acute, chronic and life‐threatening conditions.

Finally, in the third dimension, provision of care, the competency related to the implementation of therapeutic plans, interventions, treatments or therapies was prominently present, either fully or partially, in 95.83% (23) of the consultations. However, it is important to note the complete absence of elements associated with the competency related to cultural diversity.

The scene and speech excerpt presented for this dimension exemplifies these competencies, as the nurse implements a care plan to address the health demand presented, carrying out pharmacological and non‐pharmacological interventions and prescribing medications within her scope of practice in accordance with established clinical guidelines and protocols.

In summary, the results indicate that some APN‐proposed competencies were identified in the investigated NC for AE, albeit superficially in relation to the knowledge, skills and attitudes essential to their expression. Furthermore, the identified competencies were partial and scarce.

## 6 Discussion

This study aimed to describe RN practices during AE consultations in PHC settings and analyse whether RNs demonstrate the competencies proposed for APN within a care management scope. The findings indicate that while some competencies were observed, their application was often partial and inconsistent. Additionally, challenges in implementing the nursing process and maintaining effective clinical communication were noted, suggesting gaps in professional training, institutional support and standardised guidelines.

The participating RN profile presented herein aligns with Brazilian trends regarding educational background and professional experience (Gomes et al. 2024), with long‐standing professional ties to the institutions they worked in (Sousa 2022). While these RNs demonstrate a solid educational foundation and extensive professional experience, the gaps in their competency application still point to opportunities for improvement.

It is important to note that municipality C did not provide AE care, highlighting the predominance of programmatic health demands over acute care in that context. This reflects the care model adopted by the health unit, rather than a lack of competencies among the RNs. Due to Brazil's regional diversity, there is, in practical terms, no standardised approach to the processes implemented within healthcare units.

Formative, historical and cultural factors (Morelato et al. 2021) have contributed to RNs' greater familiarity and confidence in managing chronic conditions compared to handling AEs in PHC settings. A recent study (de Carvalho et al. 2024) found that most RNs identified a significant need for both theoretical and practical knowledge to manage acute demands, especially within a protocol‐based framework, highlighting the need for further training. Many of the RNs involved in the study assumed this responsibility without prior preparation, which contributed to insecurity in their practice. Another study (Canuto et al. 2021) also identified gaps in knowledge, particularly in clinical decision‐making, protocol management and the roles of RNs and their teams in managing AEs in PHC, highlighting the need for improvements in training and professional development programs.

Concerning NC and AEs, most consultations involved unscheduled visits (Canuto et al. 2021). Effectively organising PHC access to manage both scheduled and unscheduled demands remains a key challenge for management. This organisation is crucial for maintaining the core attributes of PHC and upholding SUS principles, as it is essential to not only offer planned activities but also to respond to unpredictable AEs, which represent a significant healthcare demand (Mendes 2019).

To address these challenges, health service models like advanced access (AA) have been introduced to better manage those urgent, unscheduled PHC demands, further strained by the COVID‐19 pandemic and seasonal epidemics (Canuto et al. 2021; Franco et al. 2021; Silveira 2021; Chueiri 2019). AA prioritises providing care when patients need it, addressing AEs without future scheduling, and integrating with other care pathways. Expanding nursing interventions within the APN scope offers a promising solution, improving accessibility and care quality in AE situations, while also fostering integration with longitudinal care.

Regarding the aspects of the NC process, the RNs primarily focused on biological factors (such as physiology and anatomy), often conducting superficial data collection. As a result, this frequently led to inadequate attention to the specific needs of patients (Stewart et al. 2017). Several factors, including institutional prioritisation of quantitative health production indicators, time‐constrained schedules and multi‐tasking, all contribute to hindering RNs' ability to effectively manage AE.

The findings support these observations, particularly the correlation between consultation duration and adherence to the nursing process steps. Longer consultations may offer greater opportunities for comprehensive assessments and care planning, allowing RNs to collect detailed information, address patient concerns and develop individualised care plans. Shorter consultations, on the other hand, may lead to incomplete assessments, missed diagnoses and suboptimal care, ultimately impacting patient outcomes (Tsiga et al. 2013). Another study conducted in Mexico further highlighted that patients who experienced longer consultations reported higher satisfaction levels (Torres‐Reyes et al. 2023).

Given these challenges, improving work processes is crucial. System inefficiencies can prevent RNs from fulfilling all their responsibilities, leading to pressure for quicker care. This often results in overload and stress, which in turn encourages practices focused solely on addressing immediate complaints (Bohusch et al. 2021; Norman and Tesser 2015). Moreover, in addition to gaps in training, there is a common lack of understanding among professionals and interprofessional teams about RNs roles in managing patient demands.

With regard to the nursing process, adherence and compliance to its stages are crucial during the NC, as it supports the development of clinical reasoning (Caballero 2020; Ribeiro and Padoveze 2018). The assessment stage plays a critical role, providing an initial opportunity to build rapport with the patient and explore factors related to their health concerns, including clinical manifestations, medical history, expectations and psychosocial factors. It is important to note that if this step is conducted partially, essential care aspects may be overlooked (COFEN 2024; Stewart et al. 2017), potentially leading to setbacks in subsequent steps and resulting in inadequate care plans.

The diagnosis phase of the nursing process is particularly insufficient, especially in documentation, where nursing‐specific nomenclatures are rarely used. The CIAP‐2 (WHO 2009) classification was the most commonly applied, likely due to its mandatory inclusion in the PHC Information System (e‐SUS/SISAB). However, COFEN guidelines state that the CIAP‐2 should complement, not replace, other nursing‐specific classifications (COFEN 2022). Using proper nursing diagnoses enhances the profession's identity, fosters recognition of nursing practices, and supports patients throughout their care journey.

Communication is another vital tool throughout the nursing process. Effective communication not only strengthens the nurse–patient bond but also ensures safe, qualified care, thereby improving patient satisfaction and adherence to care plans. Significant gaps in communication skills were identified, likely stemming from superficial training during nursing education (APSREDES 2018), directly influencing nursing process compliance.

A previous study highlights the challenges of implementing the nursing process in PHC settings, citing inadequate training and a lack of knowledge on the subject (Hermida and Araújo 2006). RNs perceive that their academic training does not prepare them to carry out the nursing process in a PHC context, as this is usually more hospital focused. Thus, recommendations to strengthen nursing process PHC implementation, aiming at improving nursing care effectiveness are suggested, such as situational diagnoses, team awareness, theoretical framework definitions, nursing process instrument development and practical nursing process implementation preparation through continuing education initiatives (Hermida and Araújo 2006; Almeida et al. 2023).

Some APN‐proposed competencies in PHC settings were identified in the NC presenting greater compliance with nursing process steps, albeit only partially. In general, these RNs performed better in the care dimension, which encompasses assessment skills, mainly carried out during the first nursing process step, with greater emphasis on the incorporation of knowledge concerning the clinical manifestations of normal health events, as well as of acute and chronic diseases.

However, the development of expected competencies, including for generalist nurses (RNs) working in PHC settings, such as knowledge concerning cultural diversity and health determinants and the identification of health and psychosocial risk factors, was poorly identified. This demonstrates that, in general, only information concerning biomedical diagnoses is considered essential, resulting in care plans based only on professional beliefs, which do not always represent the patient's sociocultural universe (Kumar et al. 2019).

Concerning the assessment and diagnosis dimension, the most developed competence, even if only partially, was reaching differential diagnoses between acute, chronic and life‐threatening conditions, with care proposals compatible with the identified risk. Most RNs, however, did not provide guidance on pathophysiology, expected disease evolution or warning signs/reasons for seeking new care related to the presented AE.

Regarding the care provision dimension, a competence concerning the initiation of therapeutic plans through the implementation of both pharmacological and non‐pharmacological interventions, treatments or therapies, was noted. A significant gap concerning the adaptation of these interventions to respond to the needs of people and families during life transitions and psychosocial and financial situations was, however, observed. Drug prescription was present in most NC. This item was considered as not applicable in cases where no protocols supporting the prescription to address the presented demand were available.

The findings of this study provide insights to support the discussion of APN within the Brazilian PHC context. In this regard, it is essential to emphasise that a crucial step for Advanced Practice Nursing implementation in Brazil is the definition of the core competencies for its establishment and performance, as well as its responsibilities and duties. Competence definition is paramount in the conceptualisation of this practice and can support the development of policies and regulations for its implementation (Canuto et al. 2021).

Brazil has made significant progress in the discussions surrounding Advanced Practice Nursing implementation, employing positive international experiences as references. As NC and AE exhibit a greater clinical scope, nurses expand access to quality healthcare, especially in remote regions. These practices have been developed in countries such as Australia (McCullough et al. 2020), Canada, Singapore and Taiwan (Ribeiro and Padoveze 2018), among others, with limits normally delimited by local regulatory laws, especially those that define the possibility of drug prescription (Canuto et al. 2021). A higher volume of international scientific literature on these services within hospital settings compared to PHC is, however, noted. Concerning PHC, the predominant literature evidence concerning APN is associated with prevention and promotion initiatives and the monitoring of patients with chronic conditions (Cassiani and Dias 2022; Püschel et al. 2022).

Among Latin American countries, Brazil presents one of the most favourable conditions for implementing Advanced Practice Nursing, as it has increasingly advanced the nursing teaching process, with a high number of postgraduate programs, including master's and professional degrees and residency programs (Cassiani and Dias 2022). However, certain challenges are still noted, such as the predominance of the biomedical model, the resistance of some professional categories, including doctors, and a lack of government incentives (Cassiani and Silva 2019; Cassiani and Dias 2022; Püschel et al. 2022). Furthermore, nursing practices are based on laws, policies and ordinances, and guided by clinical guidelines, protocols and other regulations established by municipal, state and federal managers, in collaboration with nursing representation entities and educational institutions (Sousa 2022). Therefore, collaboration with all these strategic partners is required, as creating an Advanced Practice Nursing initiative alone without first establishing a theoretical, political and labor basis to guide its exercise is not enough (Püschel et al. 2022; Lee et al. 2022; Nigenda et al. 2021).

It is important to emphasise that for RNs to effectively apply the expected skills during NC, they must receive adequate training with appropriate educational methodologies, as well as have access to the necessary equipment, instruments, recognition, investments and professional development opportunities (Dover et al. 2019). Additionally, well‐structured work processes that support the principles of care universality and comprehensiveness are essential, fostering a healthy work environment that enhances the execution of RNs' duties (Sousa 2022).

### 6.1 Limitations

The limitations of this study are primarily related to the sample characteristics, data collection methods and potential biases inherent to the study. The diversity in the number of NC related to AE and the heterogeneous professional profiles of participating RNs resulted in variability in the data, which may have influenced the identification of Advanced Practice Nursing competencies.

The methodological process of filming the consultations may have introduced observer bias, as the presence of cameras could have led to behavioural modifications among RNs and patients, such as increased self‐awareness, shyness or discomfort. This could have influenced communication styles, clinical decision‐making and overall interaction dynamics, potentially affecting the natural course of the consultations.

Moreover, due to the subjective nature of competency assessment, the identification of APN competencies was based on the researchers' accumulated knowledge and experience, supported by relevant elements from the literature. While this approach provided valuable insights, it is possible that interpretation bias influenced the classification of competencies, highlighting the need for additional validation through standardised competency frameworks and expert panels in future studies.

Another limitation concerns regional differences in the organisation of PHC services in Brazil. The study was conducted in a specific context, where PHC structures, protocols and professional roles may differ from those in other regions. These variations could affect the applicability of the findings to other healthcare settings with different workforce training models, patient profiles and service structures.

### 6.2 Recommendations for Further Research

Future researchers can focus on providing evidence to support strategic decision‐making in countries that are undergoing the process of implementing EPA, such as Brazil. It is crucial that these studies take into account the unique characteristics of local health systems, training models, legal and regulatory frameworks, while also considering the political, historical and cultural context of nursing practice in each country. Researchers are also encouraged to explore future research and test new methodological approaches beyond convenience sampling to increase the validity and generalizability of findings, and also implementation science methodologies to further refine APN competencies implementation process in different health systems, such as SUS.

## 7 Conclusion

This study identified limitations in NC for AEs in PHC settings, particularly regarding the execution, documentation and appropriation of the nursing process, with low compliance in several key elements. Additionally, the partial implementation of APN competencies revealed critical gaps in areas such as cultural competence and advanced assessment skills, while also highlighting promising aspects, such as improved integration of clinical knowledge and therapeutic planning. In this regard, future efforts should focus on educational programs that integrate themes like cultural competence in care provision, as well as practical training on advanced diagnostic and therapeutic techniques. Attention should be given to resources, favourable work processes, and the development of clinical guidelines that support nursing practice within this line of care to address the identified limitations.

The discussions raised in this study emphasise the importance of strengthening and expanding the scope of APN practice, which can contribute to promoting high‐quality nursing care in PHC settings, making it safer and more effective. This work also aims to support the advancement of knowledge that can inform future APN training programmes, identifying existing competencies in PHC nursing and highlighting knowledge gaps that require more investment and attention in implementing and training this role within the Brazilian health system.

Although this study does not provide a model for implementation, the results provide empirical evidence to inform policy decisions, educational programmes, nursing practices and planning for APN implementation in Brazil, such as the Ministry of Health, Ministry of Education, the Federal Nursing Council, the Brazilian Nursing Association and several specialised associations and organisations.

## Author Contributions

M.O.C., L.Y.A., A.L.V.‐V., D.B., P.A.A., C.P.B., N.V.F.S., K.G.L.R., C.S.M., M.V.M.N.: made substantial contributions to conception and design or acquisition of data or analysis and interpretation of data; involved in drafting the manuscript or revising it critically for important intellectual content; given final approval of the version to be published; and agreed to be accountable for all aspects of the work in ensuring that questions related to the accuracy or integrity of any part of the work are appropriately investigated and resolved.

## Conflicts of Interest

The authors declare no conflicts of interest.

## Data Availability

The data that support the findings of this study are available on request from the corresponding author. The data are not publicly available due to privacy or ethical restrictions.
